# Commentary: Adherence with a low-FODMAP diet in irritable bowel syndrome: are eating disorders the missing link?

**DOI:** 10.3389/fnut.2019.00136

**Published:** 2019-09-04

**Authors:** Bruno P. Chumpitazi, Ligia Alfaro-Cruz, Jasmine K. Zia, Robert J. Shulman, Margaret M. Heitkemper

**Affiliations:** ^1^Department of Pediatrics, Baylor College of Medicine, Houston, TX, United States; ^2^Children's Nutrition Research Center, United States Department of Agriculture, Houston, TX, United States; ^3^Department of Gastroenterology, Swedish Medical Center, Seattle, WA, United States; ^4^School of Nursing, Biobehavioral Nursing and Health Systems, University of Washington, Seattle, WA, United States

**Keywords:** FODMAP, irritable bowel syndrome, eating disorder, diet, adherence

A low fermentable oligosaccharide disaccharide monosaccharide and polyols (FODMAP) diet has demonstrated efficacy in ameliorating adult and pediatric irritable bowel syndrome (IBS) symptoms (1, 2). Though not formally studied, in clinical practice a traditional low FODMAP diet is applied in three phases (restriction, re-introduction, personalized maintenance) (3). Working with a dietitian knowledgeable about a low FODMAP diet is strongly recommended. There are potential relative contraindications to following a traditional low FODMAP diet given its potential effects on nutritional status, one of which includes an active eating disorder (4).

Eating disorder behavior was recently evaluated by Mari et al. using the SCOFF instrument within a population of adults with irritable bowel syndrome (IBS) who were instructed to begin a low FODMAP diet (5). The included subjects had failed previous IBS interventions (5). Based on the SCOFF results and measured adherence, the authors suggest that strict adherence to a low FODMAP diet should raise the suspicion of a possible underlying eating disorder. Though being aware of a possible eating disorder in any population is certainly important, particularly when dietary modification is being advised, we urge some discretion when making this conclusion.

There are aspects regarding diet in the IBS population that should be considered. In children, adolescents, and adults with IBS, diet exacerbates IBS symptoms in greater than 80 percent (6–8). Dietary self-perceived food intolerances are associated with increased severity of both IBS symptoms and decreased quality of life (6, 8, 9). A majority of children and adults with IBS alter their diets and/or are interested in learning which foods to avoid (9–12). In this light, some of the questions asked in the SCOFF instrument may not point toward eating disorder behavior in those with IBS as much as the perception that diet is an important factor affecting both IBS symptoms and quality of life. These questions include: “Would you say that food dominates your life?” and “Do you worry you have lost control over how much you eat?” We note that both of these questions were the two most frequently endorsed by subjects in the Mari et al. study (5). Ultimately, though not assessed, the relationship between endorsing some of the SCOFF questions and low FODMAP diet adherence in those with IBS may be less related to addressing potential eating disorder perceptions (e.g., distorted body image) or behaviors (e.g., restrictive eating) and more related to stronger motivation to address self-perceived food related IBS symptoms.

Stronger motivation and several other factors such as decision making, self-efficacy, socioeconomic contexts, cultural contexts, food access, and education have been shown to affect dietary regimen adherence (13). These and other potentially important factors were not evaluated in the Mari et al. study. Future studies of dietary regimen adherence in those with IBS may consider evaluating both these factors and the perception of food triggers as a source of symptoms (9, 13).

The use of the SCOFF instrument is an additional aspect which one may be consider when interpreting the Mari et al. study results. The SCOFF instrument is a screening questionnaire and not the gold-standard either for diagnosing an eating disorder or for determining the presence of eating disorder behavior (14). Due to the prevalence of eating disorders in the general population, the positive predictive value of the SCOFF instrument in identifying an eating disorder is less than 25% (15). To our knowledge, the SCOFF instrument has not been validated in populations where diet may play an important role (e.g., those with IBS or celiac disease). Furthermore, it has been recommended that positive results of the SCOFF instrument be followed by further evaluation (including questions regarding behavior) before referral to a mental health specialist (14). None of the subjects in the Mari et al. study were reported to have undergone further evaluation to determine whether either an eating disorder or related eating disorder behavior was present.

In conclusion, the findings by Mari et al. should spur further clinical investigation related to dietary adherence in IBS. However, given both the role of diet in IBS and the methodological approach employed, we suggest caution in interpreting the findings to suggest adherence to a low FODMAP diet is strongly associated with eating disorder behavior.

## Author Contributions

BC conceived, wrote, and revised the manuscript. The remaining authors contributed to the writing and critically revised the manuscript. All authors gave their final approval.

### Conflict of Interest Statement

The authors declare that the research was conducted in the absence of any commercial or financial relationships that could be construed as a potential conflict of interest.
